# Investigation of Extracellular Vesicle Corona Formation with Label-Free Optical Waveguide Lightmode Spectroscopy Method

**DOI:** 10.3390/bios16100550

**Published:** 2026-10-01

**Authors:** Péter Bokrossy, Beáta Szebeni, Tamás Visnovitz, Dorina Lenzinger, Éva Pállinger, Nóra Fekete, András I. Försönits, Zoltán Varga, Judith Mihály, Balázs Fórizs, Domonkos Pap, Apor Veres-Székely, Csenge Szász, Jamila Raufi, Edit I. Buzás, Attila J. Szabó, Ádám Vannay, Nóra Adányi

**Affiliations:** 1 Pediatric Center, MTA Center of Excellence, Semmelweis University, 1083 Budapest, Hungary; bokrossy.peter@phd.semmelweis.hu (P.B.); szebeni.beata@semmelweis.hu (B.S.); pap.domonkos@semmelweis.hu (D.P.); veres.sz.apor@gmail.com (A.V.-S.); szasz.csenge@semmelweis.hu (C.S.); raufi.jamila@phd.semmelweis.hu (J.R.); szabo.attila@semmelweis.hu (A.J.S.); 2 HUN-REN–SU Pediatrics and Nephrology Research Group, 1083 Budapest, Hungary; 3 Institute of Genetics, Cell- and Immunobiology, Semmelweis University, 1089 Budapest, Hungary; visnovitz.tamas@semmelweis.hu (T.V.); lenzinger.dorina@semmelweis.hu (D.L.); pallinger.eva@semmelweis.hu (É.P.); fekete.nora@semmelweis.hu (N.F.); forsonits.andras@semmelweis.hu (A.I.F.); buzas.edit@semmelweis.hu (E.I.B.); 4 Department of Plant Physiology and Molecular Plant Biology, ELTE Eötvös Loránd University, 1053 Budapest, Hungary; 5 Biological Nanochemistry Research Group, Institute of Materials and Environmental Chemistry, HUN-REN Research Centre for Natural Sciences, 1117 Budapest, Hungary; varga.zoltan@ttk.hu (Z.V.); mihaly.judith@ttk.hu (J.M.); 6 Department of Physical Chemistry and Materials Science, Budapest University of Technology and Economics, 1111 Budapest, Hungary; 7 HUN-REN Office for Supported Research Groups, Department of Biophysics and Radiation Biology, Faculty of Medicine, Semmelweis University, 1094 Budapest, Hungary; 8 Cantabio Pharmaceuticals Kft., 1149 Budapest, Hungary; bforizs@cantabio.com; 9 HUN-REN SU, Translational Extracellular Vesicles Research Group, 1089 Budapest, Hungary; 10 Insulinde BT., 1111 Budapest, Hungary; nora.adanyi@gmail.com

**Keywords:** extracellular vesicle, protein corona, biocorona, biosensor, optical waveguide lightmode spectroscopy, human serum albumin, PARK7/DJ-1

## Abstract

Extracellular vesicles (EVs) are lipid bilayer-delimited particles that are naturally released from all types of cells into the extracellular environment. During their release to the extracellular space, EVs adsorb biomolecules, collectively designated as EV corona. In this study, we developed a novel method for immobilizing EVs and assessing their capacity to bind biomolecules, using human serum albumin (HSA) as a model molecule for corona acquisition and optical waveguide lightmode spectroscopy as the analytical technique. Ten ng/mL poly-L-lysine (15–30 kDa), prepared in 42 mM TRIS buffer at pH 7.4, was used to achieve non-specific immobilization of EVs at a concentration of 4.5 × 10^10^ particles/mL, and also to provide surface passivation against proteins. The experiments were performed at 25 °C with a 100 µL/min flow rate. The optimal HSA detection range was between 1 and 100 ng/mL with EC_50_ = 2.39 ng/mL. A mathematical model was also established to determine the estimated number of HSA molecules that can be bound on the surface of EVs. In saturation experiments it was demonstrated that the magnitude of HSA bound on the surface of EVs corresponded to the theoretical estimate. After its development, the method was further tested using human plasma and a chosen protein (DJ-1) as well as non-protein corona components, specifically double-stranded DNA and a microRNA (miR494).

## 1. Introduction

Extracellular vesicles (EVs) are lipid bilayer-enclosed nanoparticles secreted by all cell types into the extracellular space [1]. Besides participating in the maintenance of cellular homeostasis, their primary role is to initiate intercellular communication by delivering their molecular cargo, which contain a myriad of bioactive molecules, including proteins, nucleic acids and lipids [2]. They play an important role in many physiological and pathophysiological processes depending on their source and target cell [3].

EVs are typically characterized based on their morphology, particle size, and the presence of surface and intraluminal protein markers, which are incorporated into the vesicular membrane during biogenesis and reflect characteristics of the source cell [4]. Following their release into the extracellular environment, EVs interact with and acquire a layer of exofacial surface-associated biomolecules from their surroundings, including proteins and nucleic acids such as DNA fragments and various RNA species, this structure is known as the EV corona or “biocorona” [5,6,7,8,9,10]. Several studies suggest that the EV corona is a dynamic complex of external cargo molecules, which are mostly acquired after secretion, and is distinct from transmembrane structures inherited from the source cell [11,12,13,14]. It has been reported that the presence and composition of the corona fundamentally alter the EVs surface properties and biological identity, influencing their uptake, biodistribution, therapeutic efficacy and may serve as indicators of their origin or possess diagnostic potential [6,7,12,15,16,17,18,19].

The EV corona and its formation have been explored using a range of analytical techniques. Transmission electron microscopy (TEM) has been employed to visualize the corona “structure”, typically described as a “patchy” layer or halo surrounding EVs [11]. Confocal microscopy and flow cytometry (FC) has been utilized to detect specific proteins on the EV surface using fluorescently labeled antibodies [6,11,20]. Zeta-potential measurements provide further insights into changes in surface charge caused by the adsorption or removal of molecules on the EV surface [10,21]. Among these methods, proteomic analysis has been most extensively used to identify EV-associated proteins in a high-throughput way; however, the highly abundant proteins can mask the detection of less abundant corona components [5,10,22].

Optical waveguide lightmode spectroscopy (OWLS) determines the resonance angle at which polarized laser light is coupled into a thin optical waveguide. When the incoupling condition is satisfied, a diffraction grating directs the light into the waveguide layer, where it propagates by total internal reflection, while the intensity of the coupled light is monitored at the waveguide edges using photodiodes. Because incoupling is a resonance phenomenon, it occurs only at specific angles that depend on the refractive index of the medium covering the waveguide. By continuously measuring these resonance angles, OWLS enables real-time determination of the effective refractive index, adlayer thickness, and surface-bound mass. Owing to the direct relationship between the resonance angle and the sample’s refractive index, OWLS provides highly sensitive, label-free detection of surface interactions and can be used to calculate layer thickness and adsorbed mass [23]. Furthermore, by immobilizing biomolecules on modified SiO_2_–TiO_2_ surfaces, OWLS can be adapted into highly specific biosensors [24]. Although it has never been applied to extracellular vesicle (EV) detection, its high sensitivity, surface selectivity, and real-time monitoring capabilities make it a promising platform for investigating biomolecular interactions on EV surfaces.

The EVs used for the in vitro and in vivo treatments unavoidably adsorb molecules present in the extracellular space onto their surface thus modifying their corona [11,25]. Human serum albumin (HSA), the most abundant protein in human plasma, constitutes approximately 40–60% of total plasma proteins [26,27]. Owing to its high abundance and the consistent observation of its association with the EV corona across multiple studies [6,16,28,29], HSA serves as an appropriate model protein for studying EV corona formation [5].

Therefore, the present study aimed to develop a novel OWLS method for investigating EV corona acquisition, using HSA as a model protein. In addition, a mathematical model describing EV–HSA interactions was established. The method was subsequently tested using human plasma and recombinant DJ-1 (Parkinson’s disease protein 7), a multifunctional protein involved in cellular protection against oxidative stress [30]. In addition, the methodology was expanded to assess the presence of non-protein corona constituents, including double-stranded DNA and microRNA.

## 2. Materials and Methods

### 2.1. EV Isolation

Cells from human peritoneal dialysate were obtained as described in our previous work [31] and cultured in collagen-coated T175 flasks (Sarstedt, Nümbrecht, Germany) in Dulbecco’s modified Eagle’s/Nutrient Mixture F-12 (11880-028, Thermo Fisher Scientific, Waltham, MA, USA) medium supplemented with 10% fetal calf serum (FCS), 1% penicillin and streptomycin (15140-122, Thermo Fisher Scientific, Waltham, MA, USA) at 37 °C in a humidified atmosphere containing 5% CO_2_.

After total cell confluency was reached, the medium was replaced with serum-free medium. After 24 h, 300 mL of supernatant were filtered through Steritop^®^ 45 mm Neck Size Millipore Express^®^ PLUS 0.22 µm PES, 250 mL (S2GPT02RE, Merck, Darmstadt, Germany). The filtrate was then concentrated to 100× using a tangential flow filtration system (TFF-Easy—tangential flow filtration EV concentrator 1, Hansa BioMed Life Sciences, Tallinn, Estonia). The concentrate was purified with qEV 70 (Izon 70 nm Gen 2 Column, Izon Science, Christchurch, New Zealand) size exclusion chromatography (SEC) column using an Izon automatic fraction collector (qEV Automatic Fraction Collector AFC, Izon Science, Christchurch, New Zealand) and then characterized and used as described below.

The remaining EVs were immediately snap-frozen in liquid nitrogen and stored in phosphate-buffered saline (PBS) (Semmelweis University, University Pharmacy, Institute of Pharmaceutical Organization, Budapest, Hungary) at −80 °C until the OWLS measurements.

Throughout the study, a single larger batch of EV preparation was used. The EVs were characterized immediately after isolation, after which the preparation was aliquoted, flash-frozen, and stored at −80 °C until use in the OWLS experiments.

### 2.2. Transmission Electron Microscopy (TEM)

For transmission electron microscopy measurements, 5 μL of the EV sample were applied to a Formvar/Carbon 200 mesh copper grid (Ted Pella Inc., Redding, CA, USA) and incubated for 2 min. The excess suspension was blotted with a piece of filter paper. Next, the grid was placed on a drop of water for 10 s, blotted, and then placed on a drop of 2% uranyl acetate (*v*/*v*) solution for 10 s. After blotting the excess solution, the grid was air-dried and viewed using a MORGAGNI 268D transmission electron microscope (FEI, Eindhoven, The Netherlands) operated at 80 kV and equipped with a Quemesa 11-megapixel bottom-mounted CCD camera (Emsis GmbH, Münster, Germany).

### 2.3. Nanoparticle Tracking Analysis (NTA) and Zeta-Potential Measurement

The size distribution and concentration of EVs were measured with NTA using a ZetaView PMX-120 instrument (Particle Metrix GmbH, Inning am Ammersee, Germany) and the result was evaluated with ZetaVIEW n 8.06.01 SP1 software. For sample dilution, PBS with a conductivity of 13.9 μS/cm was used. The applied laser wavelength was 520 nm. The measurement mode involved size distribution, conducted over two cycles at 11 positions. The analysis parameters were set as follows: maximum area of 1000, minimum area of 5, and minimum brightness of 20. The measurement temperature was maintained at 25 °C.

To assess the zeta potential of the corona-coated and native EVs, we used the same instrument and software. The samples were diluted in MQ-grade water, and measurements were carried out below 120 μS/cm conductivity at a constant 25 °C. The camera settings were changed to the following: shutter: 70, sensitivity: 85, frame rate: 30; continuous, 5 cycles, 2 positions. The samples were diluted based on previous NTA until optimal particle number (150–200 particle/frame) was reached.

### 2.4. Fourier-Transform Infrared Spectroscopy (FTIR)

The spectroscopic protein-to-lipid (P/L_spectr_) ratio reflects the purity and composition of the EV population, serving as a quality and characterization parameter [32,33]. Infrared (IR) spectra were obtained using a Varian 2000 spectrometer (Varian Inc., Palo Alto, CA, USA) equipped with an attenuated total reflection (ATR) cell (“Golden Gate” single-reflection diamond ATR unit, Specac Ltd., Orpington, UK), using 64 scans at a resolution of 2 cm^−1^. To enhance IR absorption bands, cysteamine-capped gold nanoparticles with 10 nm diameter were used enabling surface-enhanced IR spectroscopy (SEIRS), as described in [34]. Data processing, including ATR correction, buffer background spectral subtraction, and spectral evaluation, was performed using the Grams/32 6.0 software package (Galactic Inc., Tustin, CA, USA).

### 2.5. Immunogold TEM

For immunogold TEM, we employed a combination of previously published protocols with minor modifications [35,36]. A 5 µL aliquot of EV suspension was applied onto Formvar-coated nickel grids (SPI Supplies, West Chester, PA, USA). After a 10-min incubation at room temperature (RT), excess liquid was removed using filter paper. The EVs were then fixed with 4% paraformaldehyde (PFA) for 10 min at RT and rinsed three times with distilled water for 5 min each. Blocking was performed with a 2% sucrose solution in PBS (Molar Chemicals, Halásztelek, Hungary) for 1 h at RT. Primary antibodies were diluted in the same 2% sucrose/PBS solution and incubated overnight at 4 °C. Following three 5-min washes at RT with 2% sucrose solution, secondary antibodies diluted in 2% sucrose were applied for 1 h at RT. We opted for 2% sucrose instead of bovine serum albumin (BSA) to prevent non-specific protein binding to the Formvar film and to minimize potential cross-reactivity with BSA. Unbound secondary antibodies were removed with three 5-min washes at RT, followed by an additional three PBS washes to eliminate residual sucrose. The samples were post-fixed with 2% glutaraldehyde (Serva Electrophoresis GmbH, Heidelberg, Germany) and washed three times with distilled water for 5 min at RT. After immunogold labeling, positive–negative staining was performed similarly as previously described [36] but using UranyLess EM Stain (Electron Microscopy Sciences, Hatfield, PA, USA) instead of uranyl-acetate and uranyl-oxalate. Visualization was carried out using a JEOL 1010 transmission electron microscope (JEOL, Tokyo, Japan). EV diameters were measured using ImageJ v1.54g software. To detect the EV markers ALIX and CD81 [37], we used a rabbit polyclonal anti-ALIX (C-terminal) antibody (SAB4200477, Sigma-Aldrich, St. Louis, MO, USA) and a mouse monoclonal anti-CD81 antibody (MA5-13548, Invitrogen, Waltham, MA, USA). As secondary antibodies, we employed goat anti-rabbit IgG conjugated to 5 nm gold particles (G7277 Sigma-Aldrich, St. Louis, MO, USA) and goat anti-mouse IgG conjugated to 10 nm gold particles (G7652, Sigma-Aldrich, St. Louis, MO, USA).

### 2.6. Flow Cytometry (FC)

The EVs were characterized based on their protein markers using a FACS Calibur (BD Biosciences, San Jose, CA, USA) flow cytometer. Briefly, EVs were adsorbed onto the surface of formaldehyde/sulphate latex beads with a diameter of 3.8 μm (4.0 *v*/*v*%, dissolved in 100 μL of PBS; Molecular Probes, Eugene, OR, USA) and incubated overnight at 4 °C with gentle agitation. PBS containing 2% BSA served as the negative control. Samples were then incubated for 1 h in PBS with 2% BSA, followed by the addition of 300 μL of a 0.9% NaCl solution. Finally, the samples were centrifuged at 400× *g* for 10 min, then the beads were resuspended in 200 μL of a 100 mM glycerol solution and incubated at room temperature for 30 min. Before the measurement, the immobilized EVs were labeled using specific antibodies against CD9 (SC-13118, Santa Cruz Biotechnology, Dallas, TX, USA), ALIX and CD81 and the appropriate Alexa Fluor^®^ conjugated secondary antibodies (A-10680, A-21206, Invitrogen, Waltham, MA, USA). Control samples were incubated with the secondary antibodies alone to assess non-specific detection. The obtained data were analyzed with FlowJo Software v9 (Waters Corporation., Ashland, OR, USA).

### 2.7. Optical Waveguide Lightmode Spectroscopy (OWLS)

EV protein corona acquisition experiments were performed using an OWLS 210 instrument equipped with OW2400 integrated optical waveguide sensors (chips, MicroVacuum Ltd., Budapest, Hungary). The 12 mm long and 8 mm wide sensors consist of a 0.50 mm thick glass substrate slab coated with a 170–220 nm thick sol–gel single-mode waveguide layer (Si_x_Ti_1− x_O_2_, where x = 0.25 ± 0.05). The waveguide incorporates a dense, parallel grating with a period of 416.7 nm (2400 lines/mm) and an approximate depth of 20 nm. The refractive index of the waveguide layer (nF) is 1.70 ± 0.03, whereas that of the glass substrate (nS) is 1.53 [38,39]. The continuously rotated (±5°) sensor chips were illuminated with a linearly polarized He–Ne laser beam (λ = 632.8 nm). Incoupling of the incident laser beam occurs at two well-defined angles of incidence, corresponding to the transverse electric (TE) and transverse magnetic (TM) polarization modes of the laser. Consequently, four characteristic photocurrent peaks are detected on the sensor chip, with one TE and one TM peak on each side, using photodetectors positioned on both sides of the sensor. These peaks correspond to the incoupling angles αTE and αTM (Supplementary Figure S1). From these parameters the effective refractive index, adlayer thickness and bound mass per surface were calculated internally by the BioSense 3.27 software (MicroVacuum Ltd., Budapest, Hungary) [23,40]. The method development was carried out in a flow-injection analyzer (FIA) system using a Ismatec Reglo peristaltic pump (Ismatec, Wertheim, Germany) maintaining multiple flow rates, and an injector (Rheodyne, Rohnert Park, CA, USA) with 100 µL injection volume.

All of the experiments were conducted using Tris(hydroxymethyl)aminomethane hydrochloride (TRIS) buffer (02110-31-33, Reanal, Budapest, Hungary; 42 mM, pH 7.4) as a flow-through medium. For chip surface treatment, poly-L-lysine (PLL) with a molecular weight of 15–30 kDa and 70–150 kDa (P2658 and P6282, Merck, Darmstadt, Germany) was used at various concentrations. The surface treatment was carried out by injecting the PLL solution into the flow-through system. The EV samples were diluted to concentration ranging from 1.8 × 10^11^ to 4.5 × 10^10^ particle/mL for the OWLS measurements. For the examination of EV-protein binding, HSA (A1653, Merck, Germany) was used. DJ-1 was recombinantly produced by Cantabio Pharmaceuticals as described previously [41]. The presence of DJ-1 in the corona was detected using a DJ-1-specific antibody (AB18257, Abcam, Cambridge, UK).

The human blood samples (Hungarian National Blood Transfusion Service), were centrifuged twice at 2500× *g* for 15 min at RT, following the recommendations of the International Society on Thrombosis and Haemostasis, to obtain platelet-free plasma [42]. After the second centrifugation step, the absence of platelets in all plasma samples was verified using a Sysmex XE-2100 instrument (Sysmex Hungary Ltd., Budapest, Hungary). The plasma was then diluted two-fold with 0.2 µm filtered PBS. EV-depleted plasma samples were prepared as previously described [11]. Briefly, samples were first subjected to gravity-driven filtration using 5 µm and subsequently 0.8 µm filters. The filtrates were then centrifuged at 20,000× *g* for 40 min at 16 °C, followed by ultracentrifugation of the supernatant at 100,000× *g* for 16 h at 4 °C. The final supernatants were aliquoted and stored at −80 °C.

For the assessment of nucleic acid assimilation into the EV corona, two distinct molecules were utilized. First, a 132-base-pair-long double-stranded DNA fragment was generated by real-time reverse transcriptase polymerase chain reaction (qRT-PCR) using the Light Cycler 480 system (Roche Diagnostics, Penzberg, Germany) with primers specific for the DJ-1 coding *PARK7* gene (forward: 5′-TTT ATC TGA GTC GCC TAT GGT G-3′; reverse: 5′-TTT GGA TGC AAG GTC ACA AC-3′). The resulting PCR product was subsequently purified using the Monarch Spin PCR & DNA Cleanup Kit (5 µg) (#T1130S, New England Biolabs, Inc., Ipswich, MA, USA) according to the manufacturer’s instructions. As a second nucleic acid component, a custom single-stranded microRNA (miRNA) oligonucleotide with the sense sequence UGA AAC AUA CAC GGG AAA CCUC (10620310, Thermo Fisher Scientific, Waltham, MA, USA) was applied.

The mathematics behind the incoupling condition, the calculation of adlayer thickness and mass of bound material per area remain quantitatively relevant up to approximately 150–200 nm from the waveguide surface for binding of protein molecules [43]. Therefore, considering the size of EVs (~150 nm) and their low refractive index, the calculation of ng/cm^2^ can be treated as sensor response in arbitrary unit (a.u.). Further in this study ng/cm^2^ is only used for modeling EV-HSA binding. The data obtained during measurements were evaluated using BioSense 3.27 software (MicroVacuum Ltd., Budapest, Hungary) and Origin (Pro), Version 2018 (OriginLab Corporation, Northampton, MA, USA).

### 2.8. Statistical Analysis

Statistical analyses were performed using GraphPad Prism 10.0 (GraphPad Software Inc., Boston, MA, USA). Data were primarily evaluated using descriptive statistics and are presented as means ± standard deviations. Where appropriate, statistical comparisons were performed using Brown–Forsythe and Welch ANOVA followed by Dunnett’s T3 post hoc test. For the DJ-1 experiments, the data distribution was assessed using the Kolmogorov–Smirnov test prior to statistical comparison. Due to the relatively small sample sizes, variance-robust statistical methods were applied to minimize the potential influence of unequal variances on the comparisons. The reported sample size (*n*) in figure legends and methods refers to independently performed measurements using the same EV preparation.

## 3. Results and Discussion

### 3.1. Results of EV Characterization

**Figure 1:**
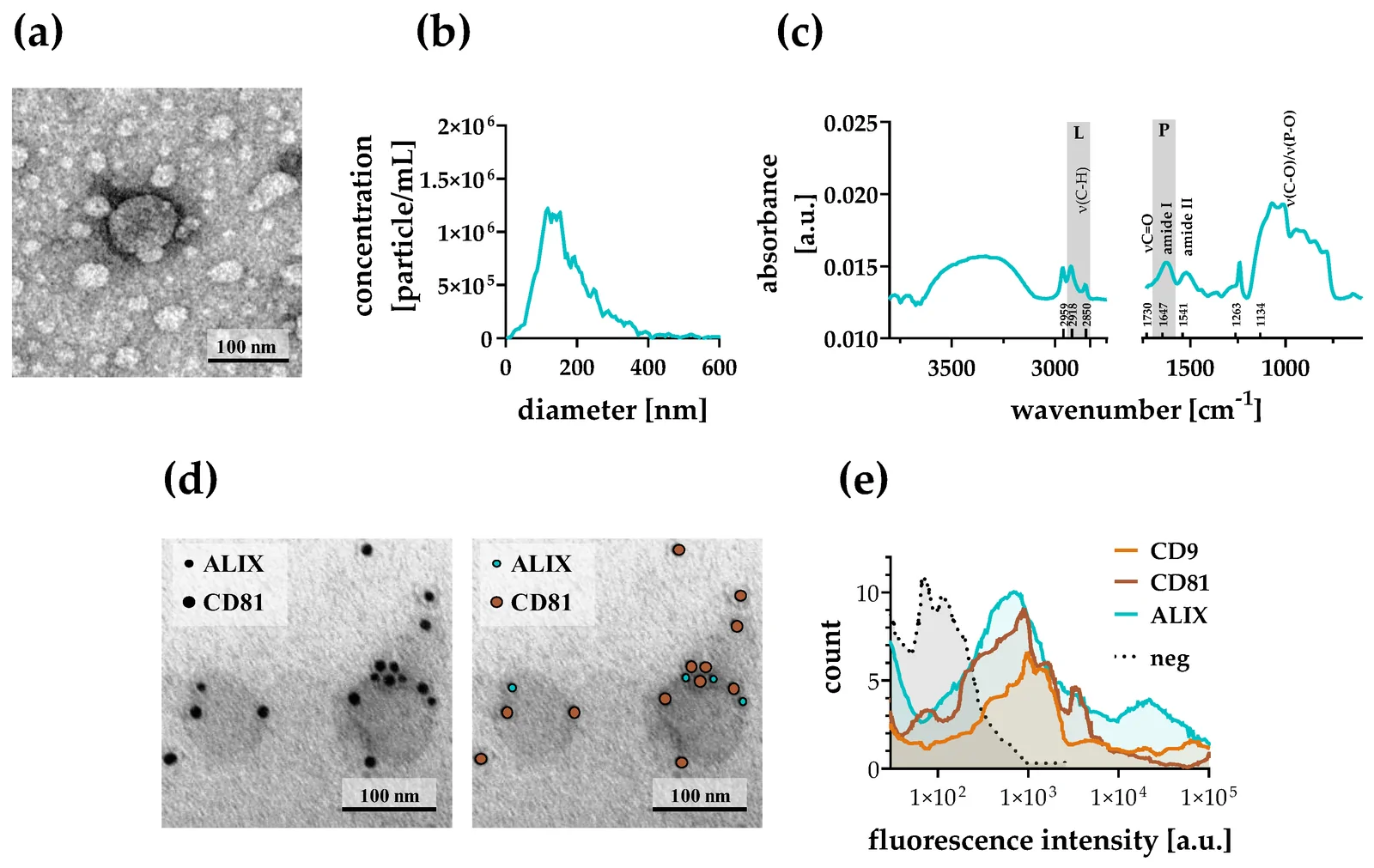
Characterization of the EVs. The EVs’ morphologies were examined with transmission electron microscopy (TEM) (**a**). Particle size distribution and concentration were determined using nanoparticle tracking analysis (NTA) (**b**). The protein and lipid content and their ratio were analyzed with surface-enhanced infrared spectroscopy (SEIRS) absorbance in arbitrary unit (a.u.) (**c**). The presence of ALIX and CD81 was determined with both immunogold TEM (5 nm gold particle—ALIX (brown) and 10 nm gold particle—CD81 (blue)) (**d**) and flow cytometry (FC) analysis (**e**), and the presence of CD9 (orange) was examined with FC (**e**). The scale bar is 100 nm on each TEM record.

The characterization of EVs was conducted in accordance with the guidelines of the International Society for Extracellular Vesicles (ISEV) [44]. Morphological characterization of EVs was performed using TEM, and the obtained recordings presented a spherical structure (Figure 1a). The NTA measurements defined the average particle size as 155 nm with a standard deviation of 26 nm and the concentration as 3.6 × 10^11^ particle/mL (Figure 1b). In the SEIRS spectrum characteristic absorption bands of EVs were observed (Figure 1c). The lipid bilayer was identified by C-H stretching vibrations of lipid acyl chains at 2921 cm^−1^ and 2852 cm^−1^, along with the C=O stretching vibration of glycerol backbone at 1737 cm^−1^. Protein content was distinguished by the amide I and amide II bands, corresponding to the peptide backbone at 1648 cm^−1^ and 1542 cm^−1^, respectively. The spectroscopic protein-to-lipid ratio (P/L_spectr_) resulted to be 2.11 ± 0.02, which is within the typical range for EVs [31,34,45]. Finally immune TEM and FC analysis revealed immune positivity for the markers CD9, CD81 and ALIX (Figure 1d,e). Based on the characterization results and ISEV guidelines our EVs can be described as small EVs.

### 3.2. Method Development and Optimization

In accordance with the literature, the acquisition of the protein corona on EVs is influenced by the environment into which the EVs are released [6,7,17]. Given that the OWLS system provides an artificial environment without the interfering effect of the surrounding biological matrix, the interaction between EVs and specific proteins can be directly studied [38,39].

It is important to note that even EVs isolated from cell culture media may carry a native corona, which can contain components derived from the culture medium. Therefore, throughout the following sections of the manuscript, the term “corona acquisition” refers specifically to the experimentally acquired corona formed on these EVs during the experiments, without addressing or characterizing the pre-existing native corona.

**Figure 2:**
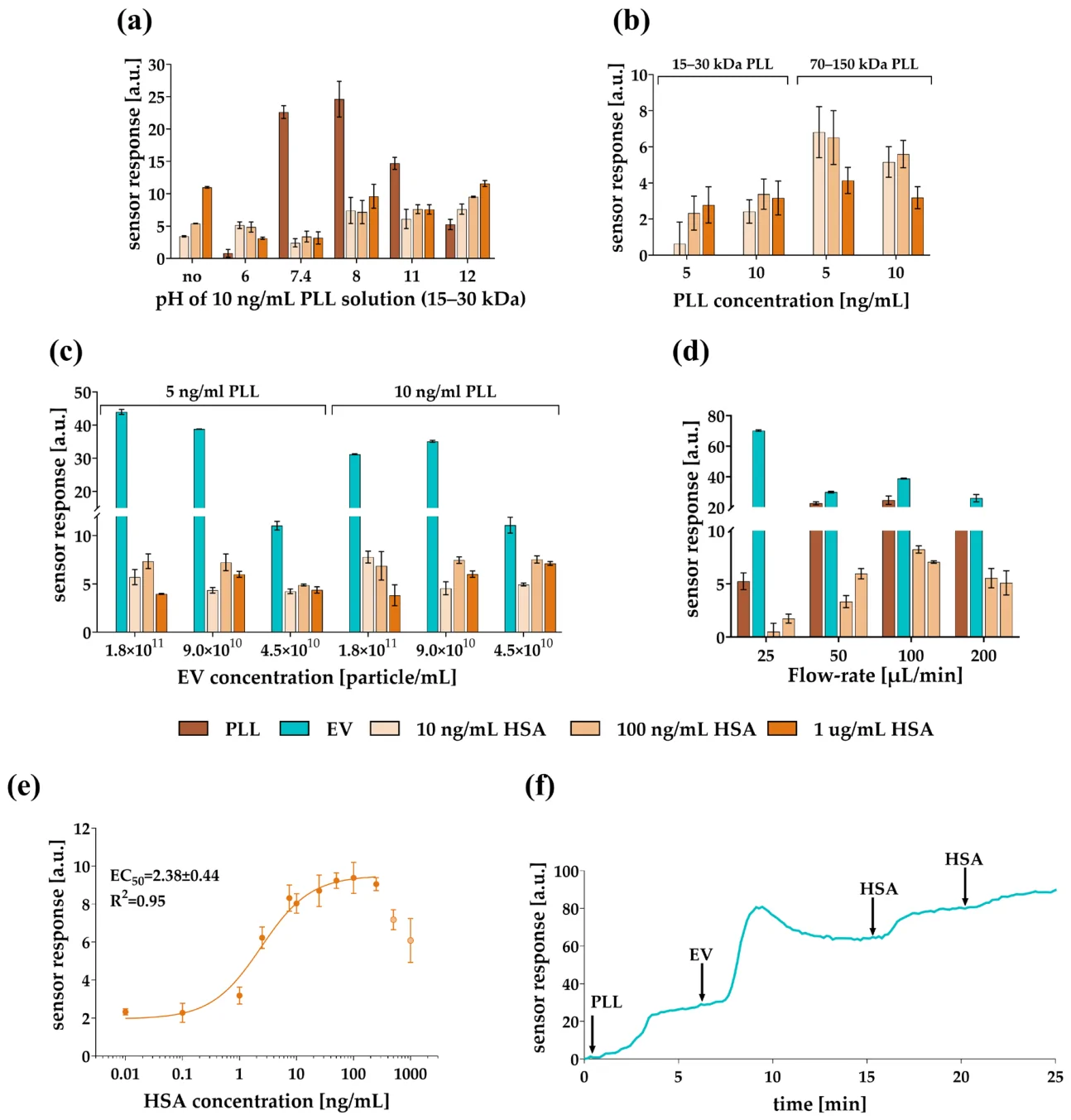
Optimization of parameters to measure EV-HSA binding by OWLS method. The pH dependency of 10 ng/mL poly-L-lysine (PLL) with a molecular weight 15–30 kDa binding to the sensor surface and its passivating efficiency for 10, 100 and 1000 ng/mL human serum albumin (HSA) (**a**). The passivating effect of 5 and 10 ng/mL PLL with molecular weights at 15–30 kDa and 70–150 kDa for 10, 100 and 1000 ng/mL HSA (**b**). The sensor response of 10, 100 and 1000 ng/mL HSA on EVs immobilized at different concentrations with 15–30 kDa on 5 and 10 ng/mL PLL (**c**). The correlation between flow rate and sensor response for 100 ng/mL HSA on 10 ng/mL PLL (15–30 kDa) immobilized EV (9 × 10^10^ particle/mL) (**d**). Concentration–response relation of HSA on 4.5 × 10^10^ particle/mL immobilized EV with a fitting of three-parameter logistic curve (EC_50_ = 2.38 ± 0.44, R^2^ = 0.95) (**e**). Sensor response as a function of time with arrows indicating injection points of PLL (15–30 kDa, 10 ng/mL, pH 7.4), EV (4.5 × 10^10^ particles/mL), and two HSA solutions (50 ng/mL), injected sequentially (**f**). Error bars: mean ± SD (*n* = 3).

Our initial observations showed that the binding of EVs to the clean, untreated glass surface was unstable. On top of that, HSA could adsorb on it, resulting in false positive signals (3.42 ± 0.1, 5.39 ± 0.03, 11.00 ± 0.14 for 10, 100 and 1000 ng/mL HSA, respectively) as Figure 2a depicts. To promote EV binding while reducing HSA binding to the glass surface, the surface was treated with PLL. According to the literature, surface passivation with PLL depends on pH, molecular weight, and concentration [40]. Therefore, we investigated the surface passivation effect of PLL (15–30 kDa) at a wide pH range. We found that the optimum condition was at pH 7.4 with sensor responses 22.63 ± 0.99, 2.41 ± 0.64, 3.38 ± 0.84, and 3.17 ± 0.94 for 10 ng/mL PLL, 10, 100 and 1000 ng/mL HSA, respectively. Either below or above this pH, the PLL was not sufficiently bound to the surface or the immobilized PLL did not adequately passivate the surface for HSA. Indeed, at pH 6.0 the responses were 0.79 ± 0.59, 5.13 ± 0.52, 4.85 ± 0.79, and 3.11 ± 0.17 while at pH 12 they were 5.25 ± 0.79, 7.62 ± 0.78, 9.53 ± 0.10 and 11.58 ± 0.46, respectively (Figure 2a). Thus, in further experiments PLL was immobilized at pH 7.4. In the next step, we investigated the effect of the molecular size of PLL on HSA binding. Indeed, both the 15–30 kDa and 70–150 kDa PLL reduced HSA binding to the sensor surface. However, 15–30 kDa PLL showed better performance compared to 70–150 kDa PLL at both 5 and 10 ng/mL concentration (Figure 2b); therefore, this lower-molecular-weight PLL was used for further experiments.

Given that the optical properties of EVs are poor and the available surface for them on PLL is finite, the measured sensor response at higher concentration is not proportional to the adlayer mass but still represents the coverage of the PLL-treated sensor surface. The optimal concentration of EVs was determined on the PLL-treated surface at pH 7.4, using different concentrations of 15–30 kDa molecular weight PLL. As Figure 2c shows, EV injected at concentrations of 9 × 10^10^ and 4.5 × 10^10^ particle/mL offered the most available binding surface for HSA using 10 ng/mL PLL.

As OWLS measurements are performed in a flow-cell, the buffer flow rate plays a key role in the observed changes. The effect of flow rate on HSA binding to EV was determined at 25, 50, 100, and 200 µL/min (Figure 2d). Lower flow rate provided decreased signals of HSA on EV (0.52 ± 0.79 and 1.74 ± 0.42, respectively), which can be a result of a nearly laminar current that created a hydrate layer on the surface, preventing EVs and proteins from binding to it, independently of the longer residence time in the cell. On the contrary, in the case of 200 µL/min, the time of residence was not enough for the molecules to bind, also resulting in a decrease in signals (5.55 ± 0.91 and 5.10 ± 1.31, respectively). Considering all the above, a flow rate of 100 µL/min gave optimal signals (7.24 ± 0.34 and 6.06 ± 0.12, respectively) and was used for further experiments.

Finally, the effect of temperature on the measurements was evaluated at 20 °C, 25 °C, and 37 °C to identify the optimal working conditions. The HSA signals on 20 °C were lower but more stable (6.53 ± 0.34 and 4.81 ± 0.12) than at 25 °C or 37 °C (8.24 ± 0.60, 7.06 ± 0.44 and 4.91 ± 1.17, 4.09 ± 0.54). On the other hand, at 37 °C, which is the physiological temperature of humans, the signals were less specific, with increased standard deviation presumably because of the less orderly layer of PLL. In subsequent measurements, the binding of HSA was studied at 25 °C, chosen as a compromise between optimal measurement and physiological conditions. This temperature yielded the highest sensor responses, as well.

Figure 2e presents the different concentrations of HSA injected always as first after EV immobilization, to show a calibration curve for HSA measurement. The optimal measuring range and statistical parameters were determined by plotting the sensor responses as a function of HSA concentration and fitting of the three-parameter logistic curve to the resulting points. The detection range can be defined between 1 and 100 ng/mL HSA, while sensor responses at higher concentrations are no longer proportional to the concentration of the solution injected, therefore concentrations above 250 ng/mL were not considered further and were excluded from the fitting.

To conclude the optimization process, the final measuring parameters were as follows: 10 ng/mL PLL (15–30 kDa) in TRIS buffer (42 mM, pH 7.4) at 25 °C, with 100 µL/min flow rate, the isolated EVs were diluted to 4.5 × 10^10^ particle/mL and 50 ng/mL HSA was injected for EV-HSA binding measurements. Figure 2f presents the desired flow chart showing the calculated data in a.u. as a function of time. Supplementary Figure S2 shows two flow charts and the sensor responses to 100 ng/mL HSA injection under optimized conditions on PLL- and PLL+EV-treated sensor surfaces.

### 3.3. Modeling of Protein Saturation on EV Surface

EVs have not previously been analyzed using the OWLS system. As described in the Section 2, the ng/cm^2^ unit remains quantitatively valid for molecular binding events occurring within approximately 150–200 nm from the sensor surface, which corresponds to the typical size range of small EVs. Therefore, we aimed to assess the magnitude of the sensor response’s reliability. Exclusively for this purpose, a simplified mathematical model was developed to estimate the maximal density of HSA molecules (per cm^2^) capable of binding to immobilized EVs within the OWLS platform under idealized conditions. Saturation experiments were subsequently performed to compare the magnitude of the experimentally observed response with this theoretical upper bound.

**Figure 3:**
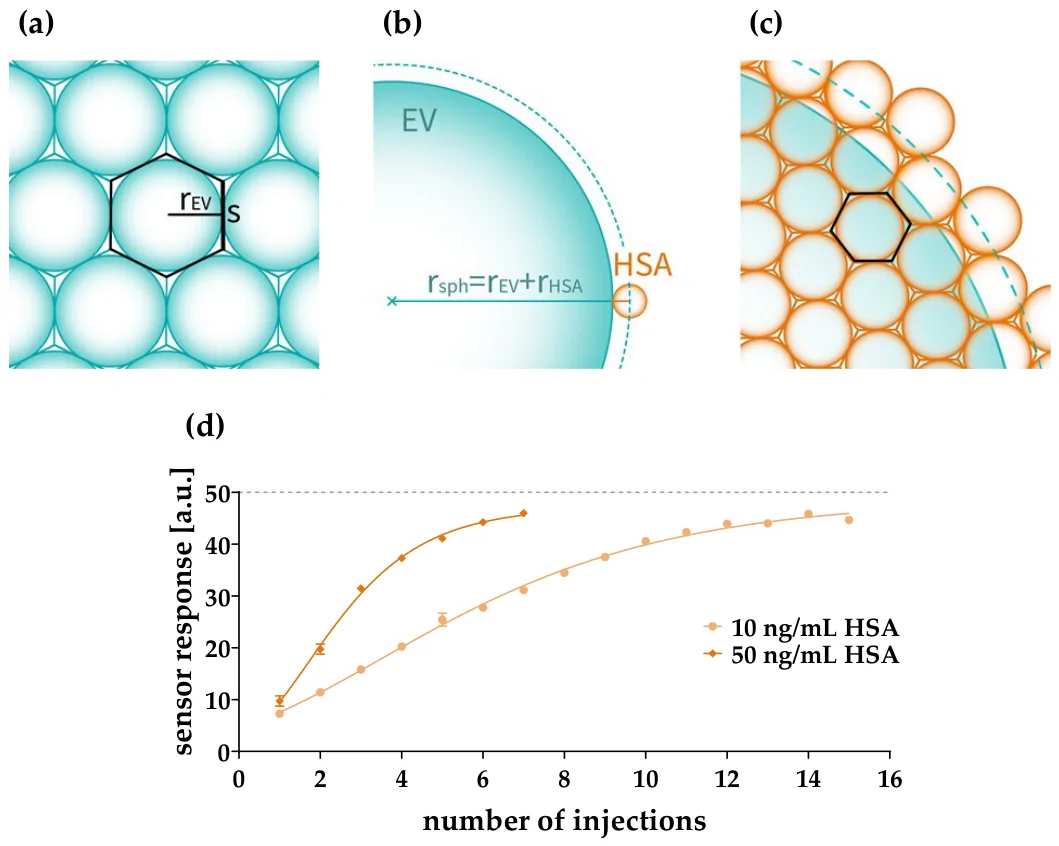
Schematic representation of the close-packed EVs (blue spheres) with hexagonal symmetry (black highlighted hexagon, and blue hexagons around EVs) (**a**), of the surface (dashed line) created by the addition of the EV and HSA (orange spheres) radii (line between the center of an EV and a HSA molecule) (**b**) and the HSA molecules tightly packed (hexagonal symmetry indicated with black highlighted hexagon and orange hexagons around HSA molecules) on this surface (**c**). Cumulated sensor response values of sequentially injected HSA solutions at 10 (light orange curve) and 50 (dark orange curve) ng/mL concentrations. Both curves converge to approximately 50 ng/cm^2^ as the dashed reference line indicates. Error bars: mean ± SD (*n* = 3) (**d**).

Previous studies have demonstrated that the negative surface charge of EVs inhibits their aggregation, therefore here we assumed that EVs form a monolayer on the waveguide surface [46]. To get some idea of the theoretical maximal number of EVs on the surface, given that our vesicles are spherical with an average diameter of 155 nm (Figure 1b) we estimated the number of EVs on the surface using Equations (1)–(3) presuming a close-packed EV monolayer with hexagonal symmetry (Figure 3a), where *s* is the length of the side of the imaginary regular hexagon around the EV, *r* is the radius of the EV (half the diameter based on NTA) and *A_EV_* is the hexagonal planar projection’s surface of a single EV.

(1) $$r=\frac{s\times \sqrt{3}}{2}\Rightarrow \;s=\frac{2r}{\sqrt{3}}$$

(2) $$A_{EV}=6\times \frac{s^{2}\times \sqrt{3}}{4}=2\times \sqrt{3}\times r^{2}=20,806\;{nm}^{2}$$

(3) $$\frac{1\;{\mathrm{cm}}^{2}}{A_{EV}}=4.81\times 10^{9}\;EV/{cm}^{2}$$

In the next step, using the hydrodynamic radius of HSA, which is approximately 6 nm [47], we estimated the theoretical maximal number of HSA molecules that can be bound to the EV surface using again a hexagonal sphere packing. Equations (1) and (2) were also used to calculate the hexagonal area dedicated to one HSA molecule (*A_HSA_*).

$$A_{HSA}=124.7\;{nm}^{2}$$

A theoretical spherical surface was assumed with a radius as a sum of the EV’s and HSA’s radii (Figure 3b), which provides a model surface where HSA molecules can be closely packed to each other with hexagonal symmetry (Figure 3c). The area of this sphere’s surface was calculated using Equations (4) and (5) where *r_HSA_* and *r_EV_* are the radii of HSA and EV, respectively, *r_sph_* is the radius and *A_sph_* is the surface of the created imaginary surface.

(4) $$r_{sph}=r_{EV}+r_{HSA}=83.5\;nm$$

(5) $$A_{sph}=4\times r_{sph}^{2}\times \pi =87,616\;{nm}^{2}$$

The maximal number of HSA (*n*) that can be packed to this surface was also calculated with Equation (6).

(6) $$n=\frac{A_{sph}}{A_{HSA}}=702\;HSA/EV$$

Under the assumption of close-packed EVs, only the upper hemisphere of EVs will be available to HSA molecules. In this sense, Equations (7) and (8) calculate the tentative number and amount of HSA on the surface of the immobilized EV monolayer on the waveguide.

(7) $$4.81\times 10^{9}EV/{cm}^{2}\times \frac{702}{2}HSA/EV=1.69\times 10^{12}\;HSA/{cm}^{2}$$

(8) $$\frac{M_{HSA}\times 1.69\times 10^{12}}{6.023\times 10^{23}}=1.87\times 10^{-7}g=187\;ng\;HSA/{cm}^{2}$$

It is important to emphasize that the mathematical model assumes idealized conditions that are unattainable in practice, both for the binding of EVs to the sensor surface and for the binding of HSA molecules to the EV surface. Furthermore, the model represents these entities as perfect spheres and does not account for any steric hindrance or charge-related interactions. Consequently, the calculated value should be regarded as a theoretical upper bound rather than an experimentally achievable saturation level, and is expected to substantially exceed the amount of HSA that can bind under real experimental conditions.

After modeling, we conducted saturation experiments on immobilized EVs with HSA to compare the results with those calculated above. In these experiments, 10 and 50 ng/mL HSA solution were repeatedly injected onto the EV-covered waveguide to approach saturation. After 15 injections of 10 ng/mL HSA solutions, the cumulated signal reached about 50 ng/cm^2^. With 50 ng/mL HSA, a cumulative signal of approximately 48 ng/cm^2^ was reached after only five injections. In both cases, the response approached a similar level despite the difference in HSA concentration, consistent with a finite EV surface area available for the proteins (Figure 3d).

The experimentally observed response was approximately of the same order of magnitude as the theoretical estimate. The mathematical model represents an idealized upper-bound scenario and therefore cannot be expected to predict the experimentally attainable saturation level. Several factors should be taken into consideration when interpreting the difference between the two values. Indeed, the theoretical maximum number of EVs that could occupy the sensor surface was estimated based on the mean particle size determined by NTA. It is important to consider that the model estimates the maximum number of HSA molecules that could bind to an EV of a fixed size in the absence of a pre-existing corona. In contrast, as shown in Figure 1, the EV population exhibits a moderate size distribution, and EVs derived from cell culture may already carry a native corona. These factors may therefore contribute, at least in part, to the lower experimental response observed compared with the theoretical estimate.

Nevertheless, the experimental results indicate that HSA binding to EVs can be detected within the applied OWLS sensing range without an apparent substantial loss of sensor response associated with the finite sensing depth. However, considering the limitations mentioned above, the OWLS response should preferably be interpreted as a sensor response reflecting changes in the surface-associated material rather than as a direct quantitative measure of the absolute surface-bound mass.

### 3.4. Confirmation of the Developed Method

Several studies indicate that alterations in the EV corona—such as its modification or removal—lead to changes in the surface charge of EVs [5,21,46]. This aligns with the fact that changes in surface charge can influence biological behavior, for example by affecting cellular uptake [10,17,48,49].

**Figure 4:**
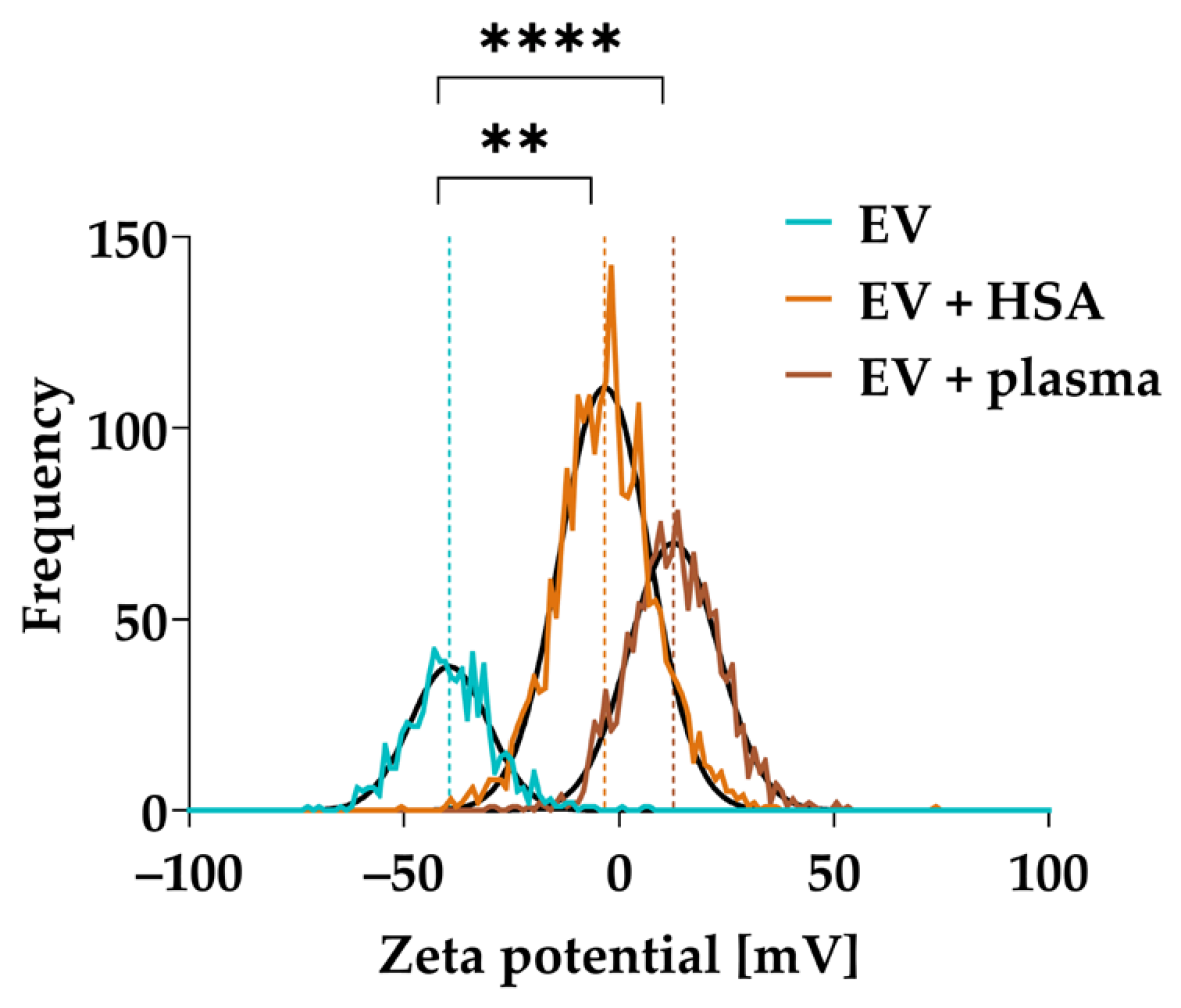
Zeta potential of EVs with different biocorona compositions. EV: vesicles with native corona (blue); EV + HSA: vesicles with corona supplemented with human serum albumin (orange); EV + plasma: vesicles with corona containing plasma-derived proteins (brown). Gaussian distribution fitting analysis is shown for each group (black). Statistical comparisons were performed using the fitted mean ± SD using Brown–Forsythe and Welch ANOVA followed by Dunnett’s T3 post hoc test. A *p*-value of ≤ 0.05 was considered indicative of statistical significance. (EV vs. EV + HSA **: *p* = 0.0012; EV vs. EV + plasma ****: *p* < 0.0001).

To support the corona acquisition observed with the OWLS system, complementary experiments were conducted. We incubated (1 h at 37 °C) the EV concentrate obtained during isolation with PBS (serving as a control), a 50 mg/mL HSA solution (mimicking HSA content of human plasma [26,27]) and EV-depleted human plasma, followed by the removal of unbound components with SEC. Subsequent changes in surface charge were assessed by zeta-potential measurements. The observed shifts presented in Figure 4 were consistent with the binding of HSA or plasma-derived components to the EV surface, as the treated samples exhibited distinct zeta-potential values compared with the PBS-treated controls.

### 3.5. Testing a Method with DJ-1 Protein and More Complex Matrix

**Figure 5:**
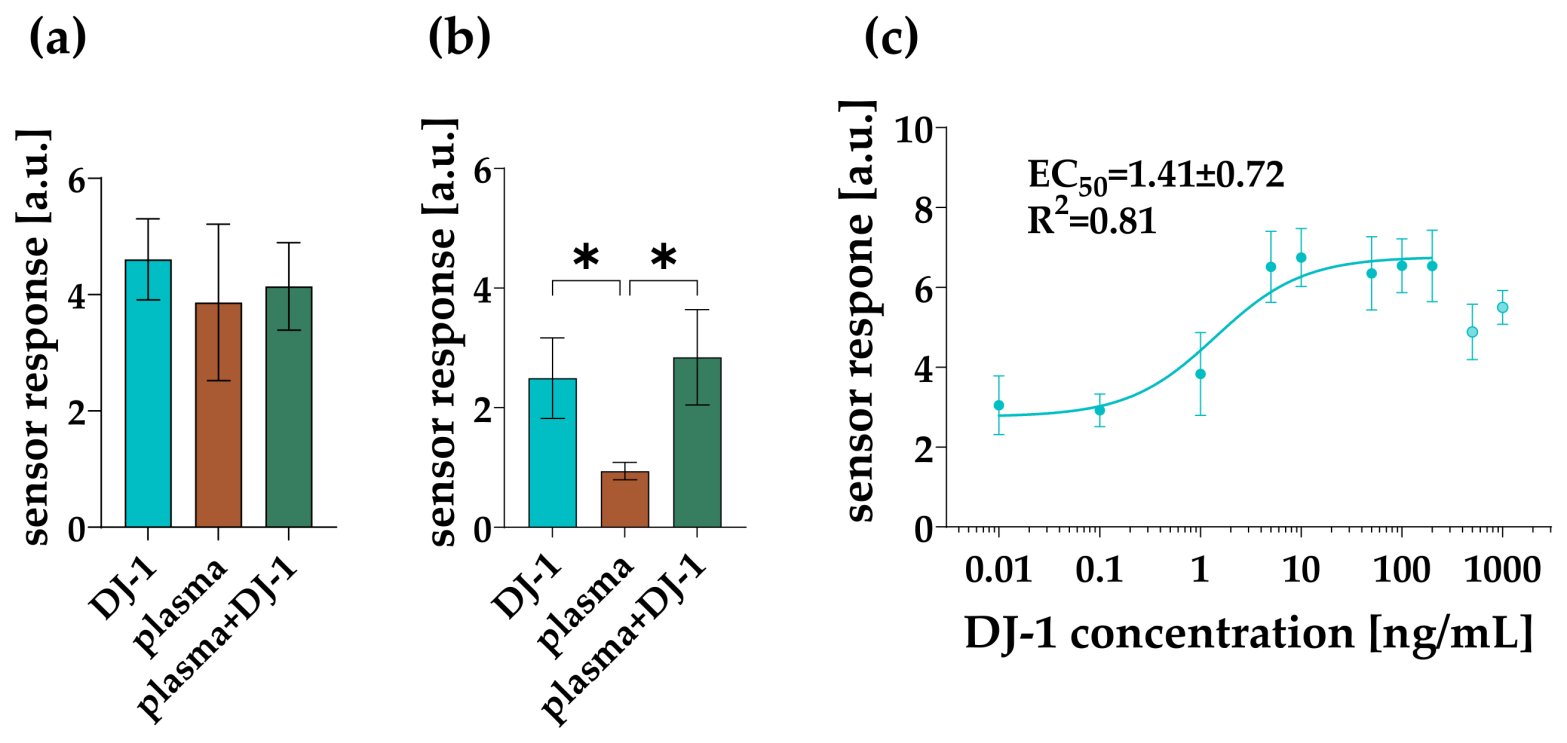
Testing of the OWLS method by investigating DJ-1 protein incorporation into EV corona from pure protein solution and complex biological matrix. Sensor responses to three consecutive injections of 100 ng/mL DJ-1 (blue), EV-depleted plasma with a total protein concentration 100 ng/mL (brown) and a plasma + DJ-1 mixture (1:1 100 ng/mL) (green) indicating protein corona acquisition (**a**). Sensor responses to three consecutive injections of 100 ng/mL antibody specific against DJ-1 protein on previously DJ-1-treated (blue), plasma-treated (brown) and plasma + DJ-1-treated (green) EVs. For statistical comparison, Brown–Forsythe and Welch ANOVA followed by Dunnett’s T3 post hoc test were applied. A *p*-value of ≤0.05 was considered indicative of statistical significance. DJ-1 vs. plasma *: *p* = 0.0464, plasma vs. plasma + DJ-1 *: *p* = 0.0202. (**b**). Concentration–response relation of DJ-1 on EV with a fitting of three-parameter logistic curve (fitting result EC50 and regression shown with errors) (**c**). Error bars: mean ± SD (*n* = 3).

The DJ-1 protein is encoded by the *PARK7* gene. Mutations in this gene have been linked to Parkinson’s disease, but the protein itself has several important functions, including protection against oxidative stress, maintaining mitochondrial balance, regulating apoptosis and autophagy, supporting dopamine synthesis and reuptake, and modulating the immune system [30,50]. In addition, in some cases it has been detected in human plasma, and its potential as a diagnostic marker in acute stroke has also been reported [51]. Given its potential presence in plasma, we identified DJ-1 as a suitable candidate to test the OWLS method established with HSA. DJ-1’s incorporation into the EV corona was evaluated using recombinant DJ-1 solution (TRIS), plasma, and a plasma + DJ-1 mixture (1:1), in each case with a final protein concentration of 100 ng/mL (Figure 5a). The presence of DJ-1 in the corona was detected using a DJ-1-specific antibody at a concentration of 100 ng/mL, applied after corona acquisition (Figure 5b). Recombinant DJ-1 served as a positive control, while plasma without added DJ-1 was used as a negative control.

Finally, similar to HSA (see Section 3.2; Figure 2e), dose–response experiments were performed with DJ-1, showing comparable EC_50_ values, whereas the fitted Top parameter was lower for the DJ-1 (Figure 5c). Thus, the two proteins exhibited similar concentration dependence in the half-maximal response region, but differed in the magnitude of the maximal measured response. This difference in response amplitude may reflect differences in the overall signal-generating capacity of the two proteins. However, based on the concentration–response data alone, it cannot be determined whether this phenomenon originates from differences in interaction stoichiometry, transport/mass-transfer characteristics, or other system-specific factors. Further investigation of the underlying mechanisms, as well as the potential biological basis and implications of this difference, is beyond the scope of the present study.

It is important to note that both the incorporation of recombinant DJ-1 and its incorporation from the plasma-containing mixture occur through a non-specific binding process. The aim of these experiments was to demonstrate that the developed method can be applied both to a defined solution containing only the molecule of interest and to a more complex biological matrix containing the same target molecule. Furthermore, given the label-free nature of the experimental setup, the presence of the specific component can subsequently be confirmed using specific antibodies.

### 3.6. Non-Protein Molecule Incorporation into EV Corona

As mentioned in the introduction, the EV corona may contain not only proteins, but also other types of molecules, such as nucleic acids [9]. Such EV-bound molecules may have diagnostic value and may also provide information about the origin or function of EVs [52].

To investigate the incorporation of nucleic acids into the EV corona, two different molecules were used. First, a double-stranded DNA PCR product was prepared using primers specific to a 132-base-pair region of the *PARK7* gene. This was followed by testing a microRNA (miRNA), miR494, which is known to downregulate DJ-1 expression. The primary aim of these experiments was to show that the developed OWLS method can also be applied to assess the incorporation of nucleic acids into the EV corona. As with the proteins studied, dose–response experiments were performed.

**Figure 6:**
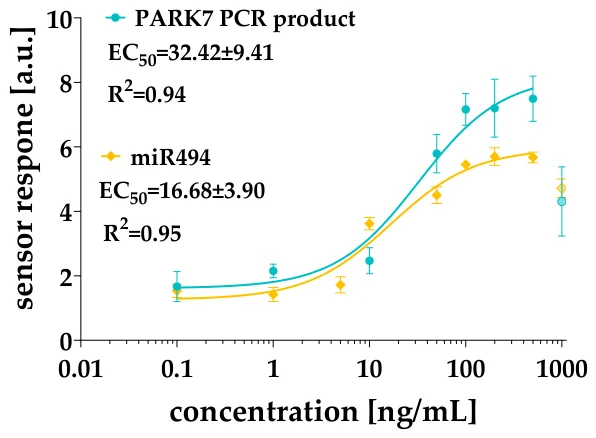
Concentration–response relation of PARK7 genes polymerase chain reaction (PCR) product and microRNA miR494 on EV with fittings of three-parameter logistic curve (fitting results EC_50_ with error bars and regression). Error bars: mean ± SD (*n* = 3).

As shown in Figure 6, both oligonucleotides were tested across a concentration range of 0.1 to 1000 ng/mL. Sigmoidal curve fitting revealed similar inflection points and comparable amplitudes. The relatively higher inflection points are not unexpected, considering the size differences between proteins and the nucleic acids used.

Given the differences in the molecular nature of nucleic acid corona components, including their structure, composition, and charge, their potential non-specific binding to the EV-free sensor surface was evaluated using both a PARK7 PCR product and miR494. The sensor responses on sensors treated with PLL only, observed for these two components, were negligible, with values of 1.08 ± 0.21 and 1.35 ± 0.53, respectively.

The overall detection limit can be estimated by defining the threshold for a detectable sensor response as three times the standard deviation of the baseline signal, with values exceeding this threshold considered indicative of a true sensor response. In addition, several binding partners were investigated in the context of EV corona acquisition in this study. For nearly all of the binding partners tested, a distinguishable sensor response could be observed between 0.1 and 1 ng/mL concentrations. This also applies to previously described components such as HSA and DJ-1.

## 4. Conclusions

EVs, after their biogenesis during their release into the extracellular space, acquire exofacial surface-associated biomolecules, forming the structure known as the EV corona. This corona significantly alters the surface properties and biological behavior of EVs. Among the proteins involved, albumin represents an appropriate model for investigating corona acquisition.

In this study, we developed and optimized a novel OWLS-based method for immobilizing EVs and assessing their capacity to bind HSA as a model for corona acquisition. We found that, following passivation of the sensor surface with 10 ng/mL PLL (15–30 kDa), the optimal measurement conditions were 25 °C with a flow rate of 100 µL/min in the presence of 42 mM TRIS buffer (pH 7.4).

After establishing the method using HSA, we investigated the incorporation of Parkinson’s disease protein 7 (DJ-1) into the EV corona in two different sample environments: a solution containing purified DJ-1 in TRIS buffer, or diluted human serum representing a more complex biological matrix. Once corona acquisition was confirmed, an anti-DJ-1 antibody was applied to verify the presence of DJ-1 on the EV surface in both cases. Because the EV corona also includes non-protein components—such as nucleic acids like double-stranded DNA and microRNA—we further investigated their attachment to the EV surface. For this purpose, we used a purified PCR product of the *PARK7* gene, along with miR-494, a microRNA known to downregulate DJ-1 expression.

It is important to consider that even EVs derived from cell culture carry a native corona, which may influence the behavior investigated throughout this study. Furthermore, EVs derived from different sources may exhibit distinct properties, interactions, and corona characteristics. Investigating such differences could therefore be scientifically relevant and represents a potential future application of the method described here. However, it should also be noted that the EV population obtained using the isolation method described in this study may comprise distinct subpopulations, and therefore, the reported average characteristics may not apply uniformly to all EVs. Different subpopulations may contribute differently to the measured signal; consequently, the observed interactions and corona acquisition should be interpreted as population-level phenomena rather than uniform properties of individual EVs.

The newly developed OWLS-based method for the non-specific immobilization of EVs enables detailed investigation of EV interactions with proteins and other biomolecules, providing a robust platform for studying molecular behavior through real-time, label-free monitoring of corona acquisition. As a versatile platform methodology, this approach establishes a foundation for examining EV corona composition and dynamics using OWLS, while also opening avenues for the development of more targeted and specialized methodologies tailored to specific research questions within the field.

## Data Availability

Data are contained within the article.

## Supplementary Materials

The following supporting information can be downloaded at: https://www.mdpi.com/article/10.3390/bios16100550/s1, Figure S1: Representative image of the 4 peaks (2-2 TE and TM) at the beginning (blue) and the ending (orange) of an optical waveguide lightmode spectroscopy (OWLS) measurement, indicating the angle shift between the two, which serves as a basis for calculation of the bound material; Figure S2: Flow chart of three 100 ng/mL HSA solutions injected consecutively onto sensor surface treated with PLL only and with PLL + EV (a). Average sensor responses for the three HSA solutions in both cases (b). Error bars: mean ± SD (n = 3).
