# Supplementary material for: Investigation of Extracellular Vesicle Corona Formation with Label-Free Optical Waveguide Lightmode Spectroscopy Method
Source: Biosensors (Basel). 2026 Oct 1;16(10):550. doi: 10.3390/bios16100550 (PMC13702521; doi:10.3390/bios16100550)
Supplement: Supplementary file 1 [file biosensors-16-00550-s001.zip › biosensors-4529426-supplementary.pdf]

1. Supplementary Materials

1.1 Measurement of incoupling angle and intensity during the OWLS

As Figure S1 represents that the measurements are performed in the four-peak mode, with the characteristic shapes of the curves clearly visible. It shows the initial (blue) and final peak (orange) profiles of a single measurement, as this representation allows the shift in the incoupling angles to be visualized despite the limitations of the graphical display (Δ sensor response ≈ 140 ng/cm^2). The goniometer is capable of detecting these shifts with a much higher resolution (7×10^{-6} degree), and the corresponding values are subsequently calculated and processed computationally by the software BioSense 3.27 software.

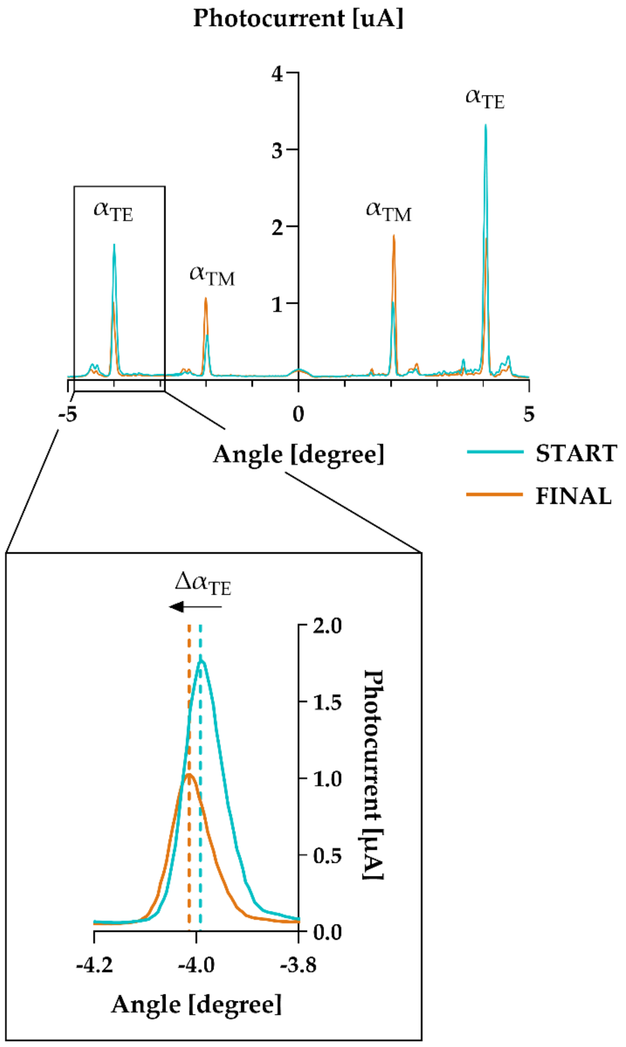

Figure S1. Representative image of the 4 peaks (2-2 TE and TM) at the beginning (blue) and the ending (orange) of an optical waveguide lightmode spectroscopy (OWLS) measurement. Indicating the angle shift between the two, which serves as a basis for calculation of the bound material.

1.2 Securing appropriate EV associated HSA signals after optimisation

Applying the optimised measurement conditions: 10 ng/mL PLL (15-30 kDa) in TRIS buffer (42 mM, pH 7.4) at 25 °C, with 100 μL/min flow rate, and 100 ng/mL HSA was applied to EV-free sensor surface and immobilised EV at 4.5×10^{10} particle/mL. As Figure S2 presents that the sensor responses for HSA comes mainly from EV-surface binding.

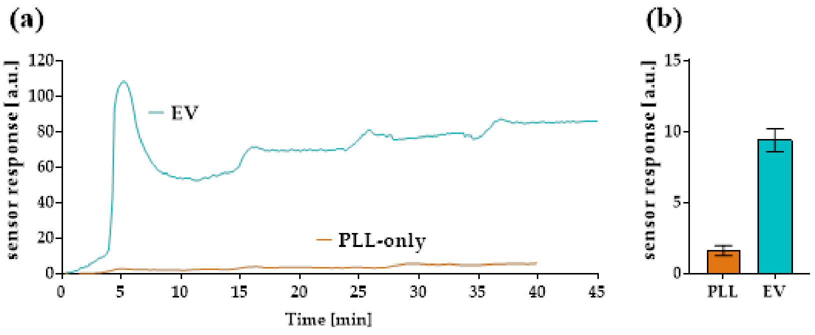

Figure S2. Flow chart of three 100 ng/mL HSA solutions injected consecutively onto sensor surface treated with PLL-only and with PLL + EV (**a**). Average sensor responses for the three HSA solutions in both cases (**b**). Error bars: mean ± SD (*n*=3).
